# Immune-related arthritis following pelvic radiation therapy in a patient with lung cancer receiving long-term immune checkpoint blocker treatment: Case report

**DOI:** 10.3389/fimmu.2022.920130

**Published:** 2022-10-24

**Authors:** Mihaela Aldea, Rakiba Belkhir, Emeline Colomba, Pierre Blanchard, Francois-Xavier Danlos, Angela Botticella, Mario Terlizzi, Eric Deutsch, Cecile Le Péchoux, David Planchard, Jean-Marie Michot, Benjamin Besse, Antonin Levy

**Affiliations:** ^1^ Department of Medical Oncology, International Center for Thoracic Cancers (CICT), Université Paris-Saclay, Gustave Roussy, Villejuif, France; ^2^ Rheumatology, Hôpital Bicêtre, Assistance Publique-Hôpitaux de Paris, FHU CARE, Le Kremlin-Bicêtre, France; ^3^ Department of Radiation Oncology, International Center for Thoracic Cancers (CICT), Université Paris-Saclay, Gustave Roussy, Villejuif, France; ^4^ INSERM U1030, Molecular Radiotherapy, Université Paris-Saclay, Villejuif, France; ^5^ DITEP (Département des Innovations Thérapeutiques et Essais Précoces), Université Paris-Saclay, Gustave Roussy, Villejuif, France

**Keywords:** radiation therapy, anti-PD(L)1, immunotherapy, toxicity, adverse (side) effects, immunoradiotherapy

## Abstract

Radiotherapy can trigger immune-related out-of-field “abscopal” response. We report a patient with advanced NSCLC (non-small cell lung cancer) receiving long-term anti-PD1 (programmed cell death protein 1) who have developed out-of-field immune-related arthritis following pelvic irradiation.

## Introduction

The “abscopal effect” was described decades ago, with the application of radiotherapy at one site leading to out-of-field response of a non-irradiated metastatic site. This phenomenon has possible underlying immune mechanisms, based on the stimulation of antigen release, immune cell recruitment, and memory (1). The synergism between radiotherapy and immune checkpoint blockers (ICBs) has shown activity, and is currently being investigated in immunoradiotherapy clinical trials (2, 3). In stage III unresectable non-small cell lung cancer (NSCLC), ICB use post curative intent concurrent thoracic chemoradiation is now the standard of care (PACIFIC trial). In the PACIFIC trial, increased severe adverse events were not reported with ICB, as compared to placebo (4).

Immune-related adverse effects (irAE) are observed with ICB; however, it remains unclear whether the addition of sequential radiotherapy after long-term ICB could promote out-of-field irAE occurrence. Here, we describe, for the first time, a potential radiation therapy-induced out-of-field irAE, in an advanced NSCLC patient undergoing long-term treatment with ICB.

## Patient information

**Figure 1:**
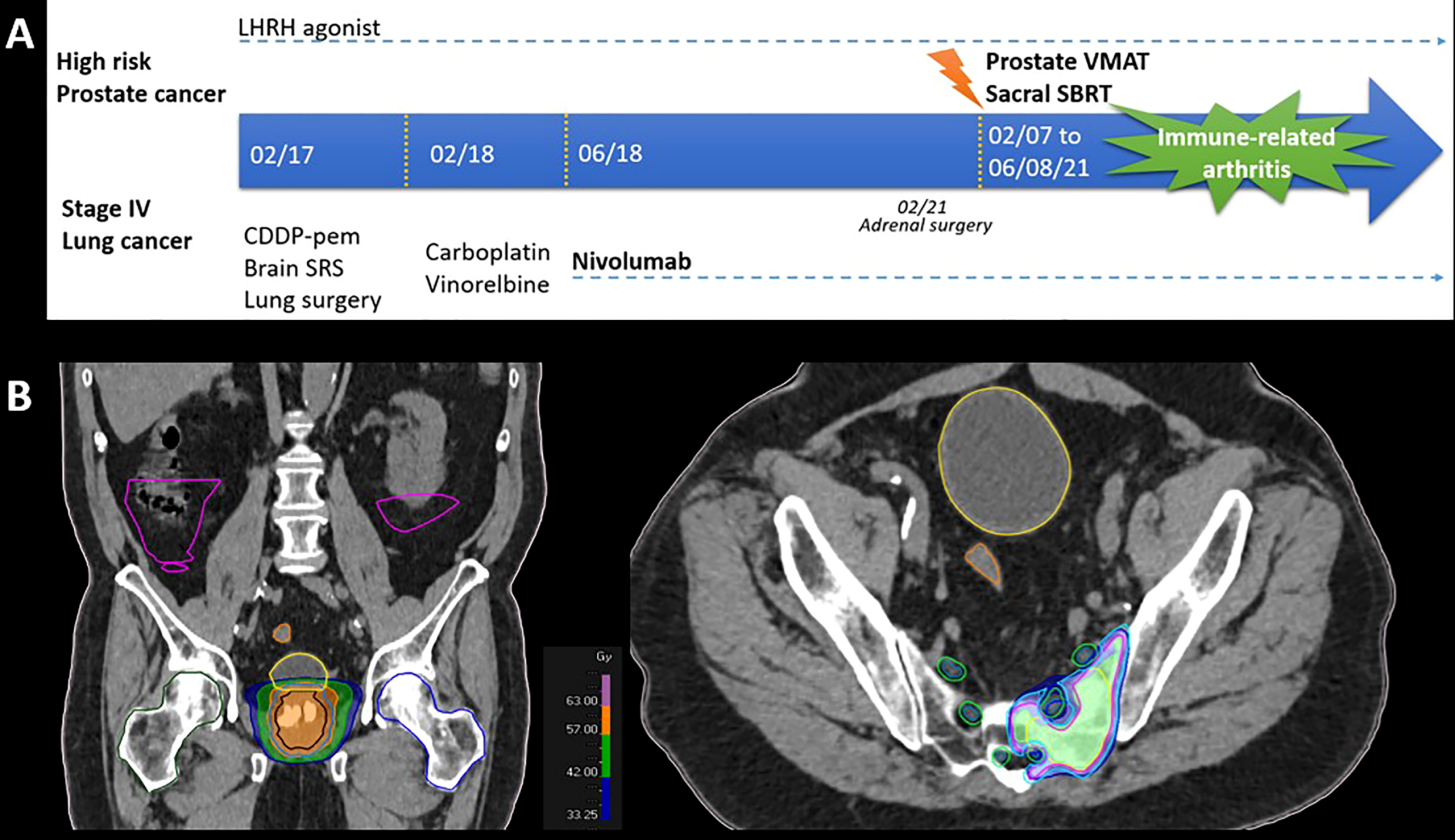
**(A)** Timelines and **(B)** radiation fields (images from the planning CT).

**Figure 2:**
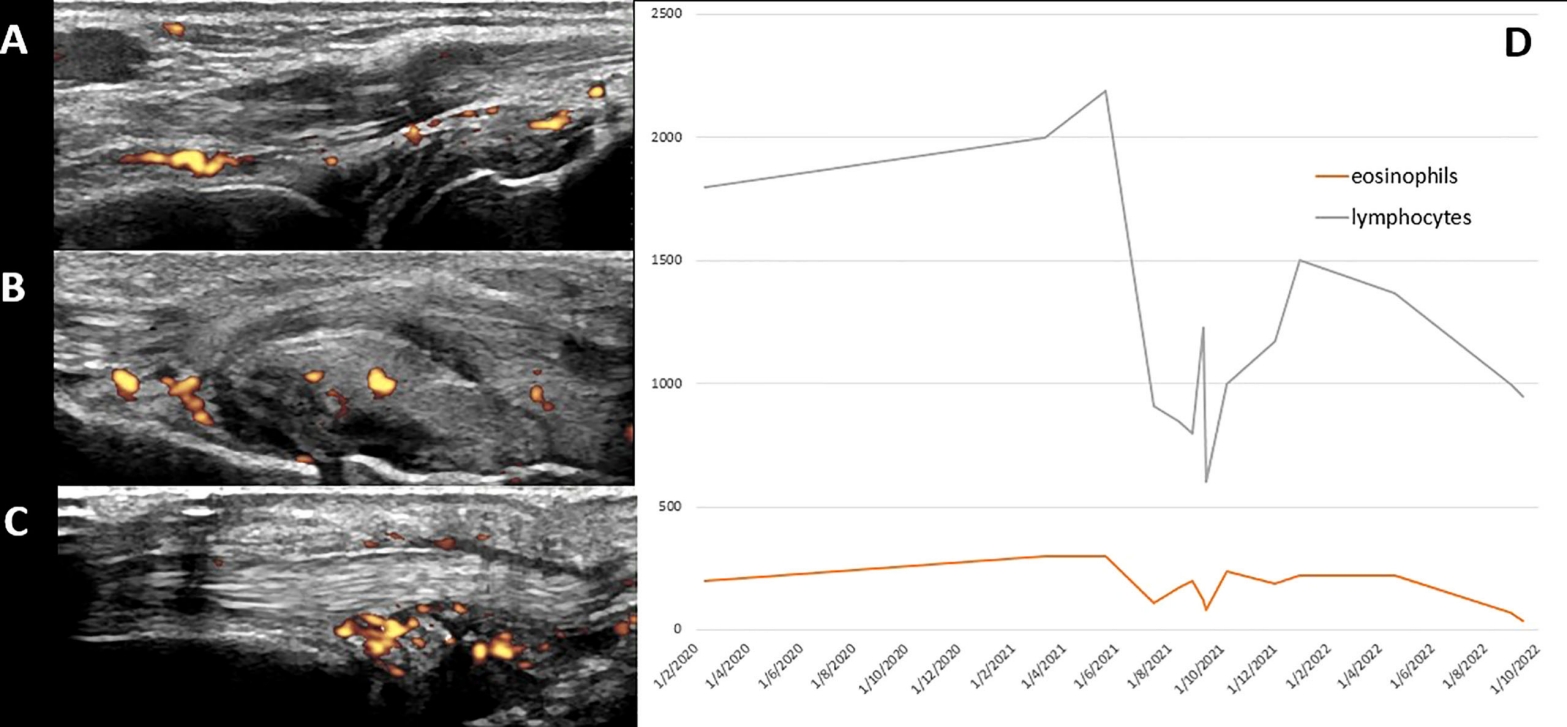
Diagnosis workup: ultrasound imaging (**A**: synovitis of the right wrist; **B**: synovitis of the right calcaneocuboid joint; **C**: tenosynovitis of the left extensor carpi ulnaris) and **(D)** plotting of blood counts.

In March 2017, a male patient in his early 70s, with a history of tobacco smoking of 30 pack-years and no personal or family medical history, was diagnosed with advanced stage IV lung adenocarcinoma (PD-L1 <1%, *CTNNB1* mutated), with two brain metastases. The patient had a synchronous locally advanced high-risk (Gleason 9, PSA = 27) prostate adenocarcinoma treated with LHRH (luteinizing hormone-releasing hormone) agonist from February 2017 (**Figure 1A**). The patient received brain stereotactic radiotherapy in May 2017. After three cycles of induction cisplatin-pemetrexed chemotherapy, he underwent a right superior lobectomy with lymphadenectomy in August 2017. In February 2018, the patient experienced oligo-recurrence with a new right adrenal and cerebellar lesion. Intravenous (IV) carboplatin (AUC 5 D1)–vinorelbine (30 mg/m^2^ D1 and D8, D1 = D21) chemotherapy was administered. However, due to progressive disease, IV nivolumab treatment (240 mg flat dose q2 weeks) commenced in June 2018. Due to an isolated adrenal oligoprogression, the patient underwent right adrenalectomy in February 2019 and continued nivolumab. In November 2020, following a significant PSA rise, choline PET-CT confirmed prostate cancer progression with a single left sacral bone metastasis. He received double site radiation therapy [prostate VMAT (volumetric modulated arc therapy) at a dose of 60 Gy in 20 fractions and sacrum stereotactic body radiotherapy (SBRT) at a dose of 35 Gy in 5 fractions] until 6 August 2021 (**Figure 1B**). From the last day of radiotherapy, he experienced invalidating poly-arthralgia predominantly of his lower limbs, with joint swelling and difficulty walking. The patient was referred to a rheumatology team. The blood samples only revealed a transient G3 lymphopenia without eosinophils variation (**Figure 2D**). Autoantibodies [including rheumatoid factor (RF), anti-citrullinated protein/peptide antibodies (ACPA), and total/soluble anti-nuclear antibodies (Anti-Ribosomes, -Sm, -SSA/Ro, - SSB/La, -U3-RNP, -centromere, -Scl-70, and -Jo-1)] were negative. Infectious analyses were negative; however, CRP was elevated (173 mg/L). X-rays were normal, joint ultrasounds revealed tenosynovitis (**Figures 2A–C**), and tendon sheath aspiration retrieved a sterile (and without crystals) inflammatory synovial fluid, with a white blood cell count of 13,000/µl (95% lymphocytes). Peripheral/central blood cultures and urine were sterile. Immune-related arthritis was suspected and steroids 20 mg/day was commenced on 27 August. Nivolumab and LHRH agonist were maintained with persistence of migrating arthralgia. Given poor efficacy of steroids, IV tocilizumab (162 mg per week) was initiated on December 2021 and symptoms improved with normalization of CRP. The patient is pursuing nivolumab and tocilizumab. His last radiological examination on 24 January 2022, including an FDG-PET/CT, showed no evolutive disease and no joint uptake.

## Discussion

To the best of our knowledge, this is the first report of a radiotherapy-triggered out-of-field irAE occurring more than 3 years after commencing ICB. Although late-onset irAEs have been described in the absence of radiotherapy, late irAEs at 3 years or higher are uncommon. Arthralgia has generally been described in approximately 10% to 15% of patients. Median time to onset of immune-related arthritis was reported to be 70 days, ranging from 1 day to 650 days (5).

Concomitant immunoradiotherapy could theoretically increase toxicities (6). First randomized reports are however reassuring with no increase of unexpected adverse events when radiotherapy is combined to ICB (2, 7). There was no significant irAE increase in the PACIFIC (consolidation immunotherapy *vs*. placebo after thoracic chemoradiotherapy) trial (4). This remains to be confirmed as real-life data suggest higher rates of observed severe toxicities (8).

Prior ICB could increase radiation therapy toxicity. Radiation recall-induced ICB could be observed up to 2 years after the end of radiotherapy, matching the previously irradiated field (9). Prior irAEs could also be linked to an increased risk of radiation therapy side effects. In an analysis of 41 patients who had prior irAEs, 61% developed subsequent ≥grade 2 radiotherapy-induced radiation pneumonitis (10).

Whether this is an out-of-field irAE or simply an incidental event after radiotherapy with unusual late onset after ICB start would require further investigation, including possible surrogate biomarkers. Unfortunately, no translational research was performed in blood or tendon sheath aspiration. The diagnosis of immune-related arthritis was suspected after other arthritis causes were eliminated and given the observed timing after irradiation. However, immune non-related adverse events such as paraneoplastic syndrome cannot be totally excluded. It is also unknown if “curative” irradiation dose, schedule, and technique may have influenced the observed immune-related arthritis occurrence. In oligometastatic-ICB treated patients, maintaining ICB beyond progression and delivering stereotactic radiotherapy to oligoprogressive sites are under investigation. Particular attention should be paid in real-life practice and in future clinical trials (e.g., NCT02756793 and NCT04405401) where ICB starts prior to localized irradiation.

## Data availability statement

The original contributions presented in the study are included in the article/supplementary material. Further inquiries can be directed to the corresponding authors.

## Ethics statement

Written informed consent was obtained from the individual AND for the publication of any potentially identifiable images or data included in this article

## Author contributions

AL and MA: Conceptualization, Data curation, Formal analysis. AL and MA: Roles/Writing - original draft. All authors: Writing - review and editing. All authors contributed to the article and approved the submitted version.

## Conflict of interest

The authors declare that the research was conducted in the absence of any commercial or financial relationships that could be construed as a potential conflict of interest.

## Publisher’s note

All claims expressed in this article are solely those of the authors and do not necessarily represent those of their affiliated organizations, or those of the publisher, the editors and the reviewers. Any product that may be evaluated in this article, or claim that may be made by its manufacturer, is not guaranteed or endorsed by the publisher.
